# Dimethyloxalylglycine Attenuates Steroid-Associated Endothelial Progenitor Cell Impairment and Osteonecrosis of the Femoral Head by Regulating the HIF-1α Signaling Pathway

**DOI:** 10.3390/biomedicines11040992

**Published:** 2023-03-23

**Authors:** Wenkai Shao, Zilin Li, Bo Wang, Song Gong, Ping Wang, Beite Song, Zhixiang Chen, Yong Feng

**Affiliations:** 1 Department of Orthopedics, Union Hospital, Tongji Medical College, Huazhong University of Science and Technology, Wuhan 430030, China; 2 Department of Rehabilitation, Wuhan No. 1 Hospital, Tongji Medical College, Huazhong University of Science and Technology, Wuhan 430030, China

**Keywords:** steroid-associated osteonecrosis of the femoral head, endothelial dysfunction, endothelial progenitor cells, HIF-1α, dimethyloxalylglycine

## Abstract

Endothelial impairment and dysfunction are closely related to the pathogenesis of steroid-associated osteonecrosis of the femoral head (SONFH). Recent studies have showed that hypoxia inducible factor-1α (HIF-1α) plays a crucial role in endothelial homeostasis maintenance. Dimethyloxalylglycine (DMOG) could suppress HIF-1 degradation and result in nucleus stabilization by repressing prolyl hydroxylase domain (PHD) enzymatic activity. Our results showed that methylprednisolone (MPS) remarkably undermined biological function of endothelial progenitor cells (EPC) by inhibiting colony formation, migration, angiogenesis, and stimulating senescence of EPCs, while DMOG treatment alleviated these effects by promoting HIF-1α signaling pathway, as evidenced by senescence-associated β-galactosidase (SA-β-Gal) staining, colony-forming unit, matrigel tube formation, and transwell assays. The levels of proteins related to angiogenesis were determined by ELISA and Western blotting. In addition, active HIF-1α bolstered the targeting and homing of endogenous EPCs to the injured endothelium in the femoral head. Histopathologically, our in vivo study showed that DMOG not only alleviated glucocorticoid-induced osteonecrosis but also promoted angiogenesis and osteogenesis in the femoral head as detected by microcomputed tomography (Micro-CT) analysis and histological staining of OCN, TRAP, and Factor Ⅷ. However, all of these effects were impaired by an HIF-1α inhibitor. These findings demonstrate that targeting HIF-1α in EPCs may constitute a novel therapeutic approach for the treatment of SONFH.

## 1. Introduction

Glucocorticoids (GCs) could be used to treat numerous disorders, including chronic sickness, severe inflammation, and autoimmune diseases [1,2]. Significantly, remarkable increases in clinical efficacy have been reported as a result of the widespread clinical application of GCs against the epidemic of viral breakouts during the peak of coronavirus sickness in 2019 (COVID-19) [3]. Osteonecrosis of the femoral head continues to be a global health hazard affecting approximately 20 million individuals [4]. Regrettably, prolonged or high-dose GCs may generate steroid-associated osteonecrosis of the femoral head (SONFH), one of the most prevalent causes of non-traumatic osteonecrosis of the femoral head. However, the pathophysiology of SONFH is not well understood and is thought to entail numerous complex pathways, including osteocyte death, homeostasis failure of osteoblasts and osteoclasts, and differentiation imbalance of mesenchymal stem cells (MSCs) [5,6,7,8,9]. The most convincing of the available theories is the vascular hypothesis, which assumes that compromised microvessels and decreased blood flow are involved in the occurrence and progression of SONFH [10]. GCs can alter the number of microvessels by inducing apoptosis in endothelial cells (ECs) and inhibiting the angiogenesis and repair capability of endothelial cell precursors, resulting in impaired and reduced local blood supply and, eventually, osteonecrosis [11,12].

Among the precursors of endothelial cells, endothelial progenitor cells (EPCs) are crucial to keeping blood vessels healthy and functioning properly [13,14]. EPCs have been shown to significantly increase the density of microvessels, promote endothelial regeneration, and improve vessel patency rates [15]. Furthermore, several studies have shown that EPCs play a role not only in the embryonic development of the vascular endothelium, but also in neovascularization and the maintenance of vascular homeostasis during adulthood [16,17]. Nevertheless, large concentrations of GCs may impair the normal biological function of EPCs. Our earlier studies have demonstrated that individuals with SONFH have a reduced quantity and function of EPCs [18,19]. Hence, it is possible to establish an effective therapeutic strategy by targeting EPCs.

As they line the inside of the vascular systems and other organs, including the femoral head, ECs play an important role in ensuring adequate blood flow, oxygen, and nutrition delivery to all of the body’s tissues under normal conditions [20]. Hypoxia, known as a state of oxygen deficiency, is thought to be one of the main pathophysiological characteristics of osteonecrosis [21]. It is hypoxia inducible factor-1α (HIF-1α) that acts as a key regulator triggered by hypoxia, which could promote EPCs dividing into ECs in the hypoxic environment. Activating HIF-1α can also enhance the expression of downstream essential target genes under hypoxia, including vascular endothelial growth factor (VEGF), stromal cell-derived factor-1 (SDF-1), transforming growth factor-β (TGF-β), platelet derived growth factor-BB (PDGF-BB), and angiopoietins-1 (Ang-1) [22,23]. The von Hippel-Lindau (VHL) and prolyl hydroxylase domain (PHD) proteins regulate HIF-1 in a constitutive manner. HIF-1 is degraded by VHL in the presence of enough oxygen after being hydroxylated by PHD [24]. Proline hydroxylase inhibitor (PHI) can promote HIF-1α stabilization and stimulate HIF-1α signaling pathway via inhibition of PHD activity [25]. Nowadays, some studies have shown that activation of HIF-1α could protect the bone from threats during physiological and pathophysiological conditions [26,27]. Activating HIF-1α in mesenchymal stem cells (MSCs) could repair SONFH by inhibiting bone-related cell damage and thereby indirectly promoting endothelial repair [28,29,30]. These findings suggest that directly targeting HIF-1α in the endothelium could be a promising alternative strategy for preventing SONFH.

Dimethyloxalylglycine (DMOG) is a permeable oxoglutarate analog that can suppress the enzymatic activity of PHD, contributing to HIF-1α degradation suppression and accumulation in the nucleus, which significantly induces downstream gene expression and can be used for the treatment of bone-associated disorders and bone tissue regeneration [31,32]. By activating the HIF-1 signaling pathway, DMOG can enhance the angiogenic potential of bone marrow- and adipose-derived MSCs, consequently boosting the amount of MSCs in peripheral blood [33,34]. In addition, exosomes produced from MSCs indirectly enhance angiogenesis and bone repair following DMOG therapy [35]. Third, the coupling of angiogenesis and osteogenesis partially mitigated bone loss produced by ovariectomy-induced osteoporosis in mice following DMOG injection [36]. Hence, we sought to determine whether immediate activation of HIF-1α by DOMG could protect against high-dose GC-related injury in EPCs.

In the current investigation, we looked at rabbit models of MPS-induced SONFH to see if DMOG treatment may reduce osteonecrosis and improve bone formation and neovascularization. Furthermore, we presumed that DMOG could improve the biological function of EPCs partially via the HIF-1a signaling pathway. Our study sheds light on a promising vascular endothelium-based strategy for the prevention and development of SONFH.

## 2. Materials and Methods

### 2.1. Cell Culture

Rabbit EPCs were isolated and cultured as described previously [37]. In brief, New Zealand white rabbits were anesthetized via ear vein injection of 10% chloral hydrate (3.5 mL/kg), and approximately 5 mL of bone marrow was withdrawn from the bilateral femur and tibia per rabbit using an aspiration needle containing 0.2 mL of heparin (3000 U/mL). The mixture was added to an equal volume of the lymphocyte separation solution (BL590; Biosharp, Wuhan, China) and centrifuged at 2000 rpm for 18 min. The resultant intermediate white flocculent cells are mononuclear cells. The cells were then rinsed twice and resuspended in M199 medium (Gibco, Carlsbad, CA, USA) containing 10% fetal bovine serum (FBS) (Gibco, Carlsbad, CA, USA), VEGF (10 µg/mL) (676491, Sigma Aldrich, Saint Louis, MO, USA), and basic fibroblast growth factor (bFGF)(2 µg/L) (PHG0026, Thermo Fisher Scientific, Waltham, MA, USA). Cells were seeded at 1 × 10^7^ cells per well in a fibronectin-coated 6-well plate, and the culture medium was changed every 48 h. On day 2, nonadherent cells were collected and seeded at 1 × 10^6^ cells per wells and cultured in M199 culture medium supplemented with 500 μM DMOG (D3695; Sigma Aldrich, Saint Louis, MO, USA) and 2% FBS in the 24-well plates for analysis of colony numbers. Control cells were treated with M199 supplemented with 2% FBS. EPC colonies were identified as elongated sprouting cells radiating from a central cluster of round cells and were enumerated blindly by two independent investigators on day 7 using an inverted microscope.

### 2.2. Characterization of Endothelial Progenitor Cells

Flow cytometric analysis (FlowJo 10.8 Software) was conducted to evaluate the EPC phenotype. After being rinsed with phosphate buffered saline (PBS) three times, the cells were digested and resuspended at a frequency of 1 × 107 cells/mL. EPCs were incubated at 37 °C for 1 h with an anti-CD34 antibody (ab81289, Abcam, Cambridge, UK; 1:200) and anti-VEGF receptor 2 (VEGFR2) antibody (sc-6251, Santa Cruz Biotechnology, Dallas, TX, USA; 1:100). To detect the number of circulating EPCs [38], 5 mL of peripheral blood from the central ear artery was put into 20 mL of red blood cell lysis solution (BL503; Biosharp, Hefei, China) containing 0.206 g Tris base and 0.749 g NH4Cl in 100 mL PBS, pH 7.2 for 10 min. After being thoroughly rinsed in MACS buffer, the cells were then treated for 15 min at 37 °C with paramagnetic microbeads (Miltenyi Biotec, Cologne, German) to remove dead cells. In a magnetic field, the cells were passed through an LS column prepared with binding buffer (MiniMACS Separator, Miltenyi Biotec, Cologne, German). The elute was incubated at 4 °C for 15 min with FcR blocking reagent and CD133-conjugated paramagnetic microbeads (Miltenyi Biotec, Cologne, German). MACS buffer was used to inject the solution into a MS column. CD133-positive cells remained in the column, while CD133-negative cells were eluted. EPCs were then incubated in the dark for 30 min with FITC-conjugated donkey anti-rabbit IgG (406403, Biolegend, San Diego, CA, USA; 1:200) or PE-conjugated donkey anti-mouse IgG antibody (406607, Biolegend, San Diego, CA, USA; 1:200), rinsed three times with PBS, and resuspended in 200 μL of PBS. The fluorescence intensity of the cells was evaluated on a FACSCalibur flow cytometer (BD Biosciences, San Jose, CA, USA).

### 2.3. Cell Viability Assay

EPCs were seeded at an initial density of 2 × 10^3^/well in 96-well plates and cultured with M199 medium containing 10% FBS in a 37 °C incubator for 48 h. Cell counting kit-8 (CCK-8) assay (CK04, Dojindo, Tokyo, Japan) was carried out to estimate cell viability after MPS treatment at different concentrations (0, 1, 10, 100, 1000 μM) for various time points (24, 48, 72 h) or after treatment with 100 μM MPS and different concentrations of DMOG (0, 50, 100, 200, 500, 1000 μM) for 48 h. Then, 10 μL CCK-8 reagent was added to each well, followed by incubation at 37 °C for 2 h. A microplate reader (Thermo Fisher Scientific, Waltham, MA, USA) was used to measure the absorbance at 450 nm and the optical density (OD) value to evaluate the cell viability.

### 2.4. Senescence-Associated β-Galactosidase Assay

A senescence-associated β-galactosidase (SA-β-Gal) staining kit (C0602, BeyoTime Biotechnology, Beijing, China) was used to assess the β-galactosidase activity of DMOG on EPCs based on the manufacturer’s protocol. After treatment for 7 days, they were fixed for 15 min with 4% paraformaldehyde and stained with the working solution, overnight at 37 °C.

### 2.5. Colony-Forming Unit Assay

For colony-forming unit (CFU) assays, EPCs derived from bone marrow cells were put in a T25 culture flask with M199 culture medium supplemented with 2% FBS. EPCs were pretreated with 500 μM DMOG alone or with 500 μM DMOG and the HIF-1α inhibitor KC7F2 (40 μM) (S7946, Selleck, Houston, TX, USA) for 24 h before MPS administration. The control group was cultured in only M199 medium supplemented with 2% FBS containing equal amounts of DMSO before MPS administration. The CFUs of the EPCs were counted on day 7. A colony of >50 cells was marked as one unit.

### 2.6. Matrigel Tube Formation Assay

The tube formation assay was performed according to the Matrigel protocol (BD Biosciences, USA). In brief, EPCs were pretreated with 500 μM DMOG alone or with 500 μM DMOG and the HIF-1α inhibitor KC7F2 (40 μM) for 24 h before MPS administration. A density of 1 × 10⁴ cells were then seeded on Matrigel for 18 h. Live cells were stained with calcein (ab141420, Abcam, Cambridge, UK), the tube formation ability of EPCs was assessed under a microscope (Olympus, Tokyo, Japan), and the total line length was quantified using the Image J software.

### 2.7. Transwell Assay

To assess the chemotaxis of stimulated EPCs, 5 × 10⁴ EPCs were pretreated with 500 μM DMOG alone or with 500 μM DMOG and the HIF-1α inhibitor KC7F2 (40 μM) for 24 h before MPS administration. Then the cells were seeded into a Transwell upper chamber (8-μm pores; Corning Co, Corning, NY, USA). A medium containing VEGF (5 ng/mL) was added to the lower chamber. Cells were cultivated for 24 h at 37 °C, and nuclei were stained with DAPI.

### 2.8. ELISA and Detection of Nitric Oxide Secretion

EPCs were cultured with 500 μM DMOG alone or with 500 μM DMOG and the HIF-1α inhibitor KC7F2 (10 μM) for 24 h before MPS administration. The control group was incubated with M199 medium supplemented with 2% FBS containing equal amounts of DMSO. VEGF and SDF-1 concentrations from the supernatant culture medium were obtained and measured using a rabbit VEGF ELISA kit (SEA143Rb, Cloud-Clone, Wuhan, China) and SDF-1 ELSIA kit (ab100637, Abcam, Cambridge), respectively. According to the manufacturer’s instructions (AKNM005M; Boxbio, Beijing, China), the supernatant of the cell culture medium was collected to detect nitric oxide (NO) levels.

### 2.9. Western Blotting Analysis

The proteins of EPCs were lysed in RIPA buffer (SW104, Sevenbio, Beijing, China) containing a protease inhibitor cocktail and then centrifuged for 15 min at 12,000× *g* at 4 °C. Proteins (20 μg per lane) were separated through 10% sodium dodecyl sulfate polyacrylaminde gel electrophoresis (SDS-PAGE) and then transferred to polyvinylidene fluoride (PVDF) membranes (0.45 μm; Millipore, USA) for analysis. After being blocked with 5% non-fat milk, the membranes were incubated with primary antibodies HIF-1α (ab179483, Abcam, Cambridge, UK; 1:1000) and β-actin (BM0627, Boster, Wuhan, China; 1:2000) overnight at 4 °C and then incubated for 1 h at 37 °C with secondary antibodies (G1213, Servicebio, Wuhan, China; 1:5000). The proteins were visualized using a Bio-Rad scanner after preparation with an ECL substrate kit (32106, Thermo Pierce, Waltham, MA, USA).

### 2.10. Animal Experiment

The Experimental Animal Ethics Committee of Tongji Medical College, Huazhong University of Science and Technology, Wuhan, China, approved all animal experimental procedures (No. S2871). Thirty New Zealand white rabbits (4 months old, body weight 3–4 kg) were used in this study. All animals were placed in hygienic plastic cages maintained at 24 °C in a clean, well-ventilated environment, with free access to normal food and water, as well as with 12:12-h light/dark cycles. To establish the GC-induced ONFH model, thirty rabbits were intravenously injected with lipopolysaccharide (LPS) (10 μg/kg; SMB00610, Saint Louis, MO, USA) via the auricular vein for two consecutive days, and three injections of MPS (Pfizer, New York, NY, USA) at 20 mg/kg body weight were administered intramuscularly at a time interval of 24 h for each group. One month after the first injection, all rabbits were randomly divided into three groups: MPS+DMSO (*n* = 10), MPS+DMOG (*n* = 10), and MPS+DMOG+KC7F2 (*n* = 10). Rabbits in the MPS+DMOG group received intramuscular injections of DMOG (20 mg/kg) every other day for 1 month. Rabbits in the MPS+DMOG+KC7F2 group were intramuscularly injected with KC7F2 (10 mg/kg) 2 h before every DMOG injection. The KC7F2 dosing regimen was based on a previous study [39]. Rabbits in the MPS + DMSO group were used as controls. All animals were sacrificed eight weeks after the first injection.

### 2.11. Micro-CT Analysis

All animals were sacrificed, and the femoral heads were collected and fixed in 4% paraformaldehyde (pH 7.4) for 3 days. Then the samples were subjected to micro-CT scanning by software (μCT 40, Scanco 274 Medical, Switzerland) at a resolution of 10.5 μm, 100 kV, and 98 μA. Images of the femoral heads were reconstructed by NRecon software, and analyzed by CTAn software to determine bone volume per tissue volume (BV/TV), trabecular thickness (Tb. Th), and trabecular separation (Tb. Sp). The cortical shell was excluded from the analysis.

### 2.12. Histological and Immunohistochemical Staining

After micro-CT scanning, the samples were decalcified with 10% ethylene diamine tetraacetic acid (EDTA)(pH 7.4) for approximately 30 days. The specimens were embedded in paraffin and cut along the coronal plane into 5-μm-thick sections. Osteonecrosis was detected by hematoxylin and eosin (H&E) staining of the sections. Osteonecrosis is characterized by the presence of lacunae or pyknotic nuclei between the trabecular bone and marrow, as well as hypertrophy of adipocytes and vascular thrombosis. Sections were classified as having osteonecrosis if they contained at least one of these features. Toluidine blue and osteocalcin (OCN) staining were performed to detect newly generated bone tissue around the osteonecrotic area. Tratrate-resistant acid phosphatase (TRAP) staining kit (G1492, Solarbio, Beijing, China) was performed to evaluate bone resorption activity. Other sections were deparaffinized, antigen retrieved, and incubated with anti-OCN (ab93876, Abcam, Cambridge, UK; 1:100), anti-HIF-1α (ab179483, Abcam, Cambridge, UK; 1:100) and anti-CD34 (ab8158, Abcam, Cambridge, UK; 1:100) primary antibodies, and then incubated with the HRP-conjugated goat anti-rabbit (GB23303, Servicebio, Wuhan, China; 1:200) and goat anti-rat (GB23302, Servicebio, Wuhan, China; 1:200) secondary antibodies. Sections were stained with DAB and counterstained with hematoxylin. All stained sections were imaged using a microscope (IX71, Olympus Corporation, Tokyo, Japan).

### 2.13. Immunofluorescence Staining

To assess neovascularization around the osteonecrotic area, we used the Factor Ⅷ as a phenotypic marker for endothelium [39,40]. Deparaffinized sections of the femur were processed using 0.25% trypsin antigen retrieval and blocked with 10% FBS for 1 h at 37 °C. The sections were incubated with primary antibody anti-Factor Ⅷ (orb381895, Biorbyt, Cambridge, UK; 1:200) at 4 °C overnight and then incubated with HRP conjugated goat anti-rabbit (GB23303, Servicebio, Wuhan, China; 1:200) secondary antibody for 1 h. Images were captured using a fluorescence microscope (IX71, Olympus Corporation, Tokyo, Japan).

### 2.14. Statistical Analysis

All data analyses were performed using GraphPad Prism 9.0 software (USA) and presented as mean ± standard deviation (SD) of at least three independent experiments. Student’s two-tailed *t*-test was performed to evaluate differences in numerical data between two groups. One-way ANOVA with the Bonferroni’s post hoc test was used to determine differences among groups. Statistical significance was set at *p* < 0.05.

## 3. Results

### 3.1. DMOG Essentially Attenuates the Cytotoxicity of MPS on EPCs

**Figure 1:**
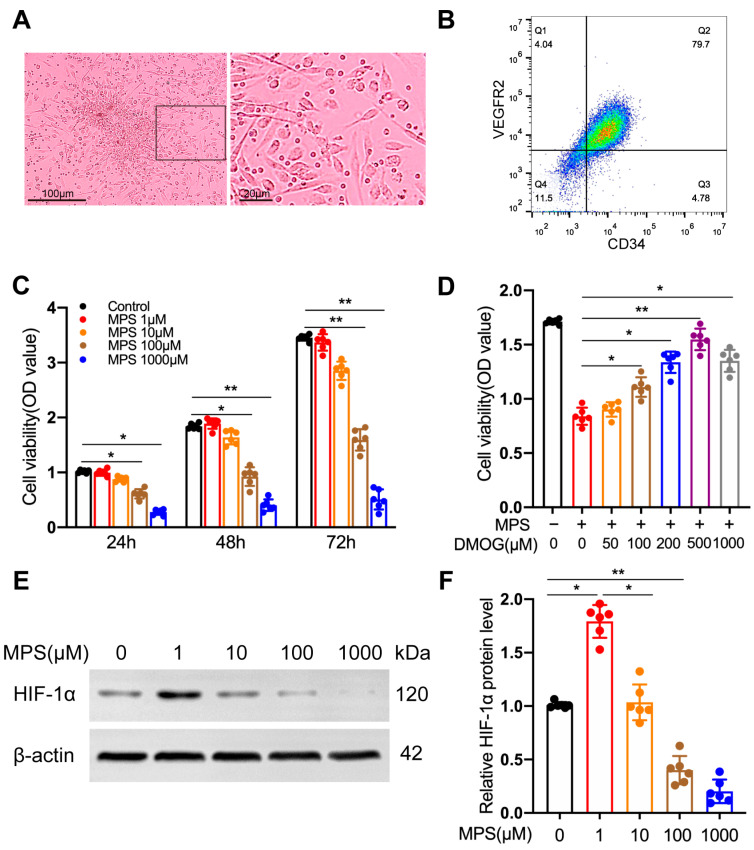
DMOG alleviates glucocorticoid-induced cytotoxicity in EPCs. (**A**) Representative cell morphology of EPCs under the light source field. (**B**) Flow cytometry analysis showed that EPCs positively expressed VEGFR2 and CD34. (**C**) The cytotoxicity of MPS at various concentrations and different time points was evaluated by the CCK-8 assay. (**D**) DMOG significantly attenuated the cytotoxicity of MPS (100 μM) on EPCs at different concentrations (0, 50, 100, 200, 500, and 1000 μM) after treatment for 48 h (**E**,**F**) Representative images of Western blotting and quantitative analysis of HIF-1α protein levels in EPCs treated with MPS for 48 h at concentrations of 0, 1, 10, 100, and 1000 μM. *n* ≥ six per group. * p < 0.05, ** p < 0.01. All data are presented as means ± SD. Statistical significance was determined by the two-tailed Student’s *t*-test.

EPCs are widely used to treat vascular disorders because of their ability to repair vascular injury and promote angiogenesis [41]. Cells in early EPC colonies were predominantly spindle-shaped, with a few spherical cells in the center after a week of culture (Figure 1A). The results of cytometric analysis showed that EPCs positively expressed CD34 and VEGFR2, confirming that we successfully obtained EPCs with high purity for further studies (Figure 1B).

To investigate the proliferation of MPS on EPCs and evaluate whether DMOG could attenuate the cytotoxicity of MPS, the CCK-8 assay was conducted to examine the effect of different concentrations of MPS (0, 1, 10, 100, and 1000 μM) on EPC viability at various treatment durations (24, 48, and 72 h). The results revealed that MPS had a dose-dependent effect on EPC viability, with a strong effect at a concentration of 100 μM after 48 h of treatment (Figure 1C). EPCs were pretreated with different concentrations of DMOG (0, 50, 100, 200, 500, and 1000 μM) for 24 h prior to incubation with MPS (100 μM) for 48 h. The results showed that DMOG decreased the cytotoxicity of MPS on EPCs in a dose-dependent manner at concentrations below 1000 μM, with 500 μM as the optimum concentration (Figure 1D).

### 3.2. DMOG Significantly Attenuates the Inhibitory Effect of MPS on HIF-1α in EPCs

**Figure 2:**
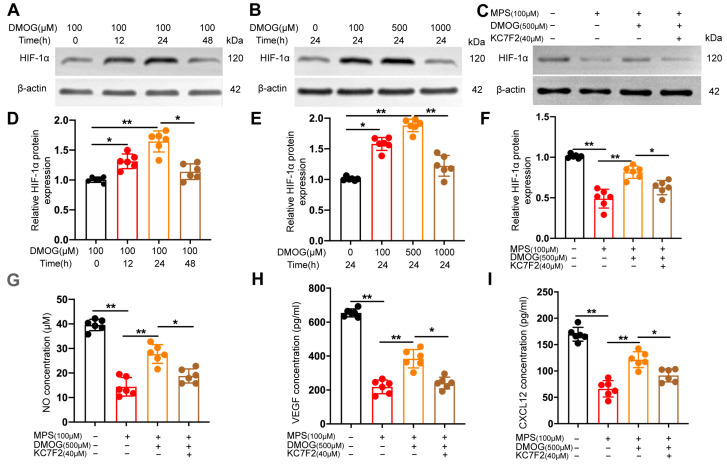
HIF-1α activation is warranted for DMOG-mediated anti-glucocorticoids effects on EPCs. (**A**,**D**) Representative images of Western blotting and quantitative analysis of HIF-1α protein levels in EPCs treated with DMOG (100 μM) for 0, 12, 24, 48 h. (**B**,**E**) The protein levels of HIF-1α were evaluated with DMOG treatment for 24 h at concentrations of 0, 100, 500, and 1000 μM. (**C**,**F**) Representative images of Western blotting and quantitative analysis of HIF-1α protein levels in EPCs pretreated with DMOG (500 μM) only or DMOG combined with KC7F2 (40 μM) for 24 h before MPS (100 μM) administration. (**G**–**I**) NO, VEGF, and CXCL12 expression levels in the culture medium supernatant as analyzed by ELISA. *n* ≥ six per group. * p < 0.05, ** p < 0.01. All data are presented as means ± SD. Statistical significance was determined by the two-tailed Student’s *t*-test.

Considering the potential effect of DMOG in the treatment of MPS-induced cytotoxicity on EPCs, our results further showed that MPS at 1 μM upregulated HIF-1α protein expression, whereas MPS (10, 100, and 1000 μM) significantly downregulated the expression of HIF-1α at 48 h (Figure 1E,F), as evidenced by Western blotting. To explore the effect of DMOG on the HIF-1α pathway, EPCs were treated with 100 μM DMOG for different timepoints (0, 12, 24, and 48 h). The results revealed that the HIF-1α protein level increased in a time-dependent manner up to 24 h, but a slight decrease occurred at 48 h (Figure 2A,D). Various concentrations of DMOG were used to evaluate the optimal concentration of DMOG in EPCs. The results showed that 500 μM DMOG significantly increased the HIF-1α protein level at the same time point of 24 h (Figure 2B,E).

### 3.3. HIF-1α Activation Is Required for DMOG-Mediated Anti-MPS Effects on EPCs

Our results showed that DMOG alleviated HIF-1α protein degradation, thereby increasing the level of HIF-1α protein. Nevertheless, EPCs chemotactic migration to injured tissues and vessels must be mediated by chemokines and other secreted factors. To further elucidate whether these effects of HIF-1α activation were a result of DMOG-mediated anti-MPS in EPCs, we used an HIF-1α inhibitor to suppress protein expression. Western blotting results showed that KC7F2 at 40 μM repressed the HIF-1α protein levels elevated by DMOG (Figure 2C,F). Next, ELISA was performed to detect the secretion levels of VEGF, SDF-1, and NO, which are downstream of HIF-1α and play significant roles in promoting both the mobilization and homing of EPCs. Pretreatment of EPCs with DMOG largely attenuated the inhibitory effects of MPS on VEGF, SDF-1, and NO secretion, whereas the addition of KC7F2 impaired the elevated levels of all these factors (Figure 2G–I).

### 3.4. DMOG Alleviates MPS-Induced Biological Disfunctions via HIF-1α in EPCs

**Figure 3:**
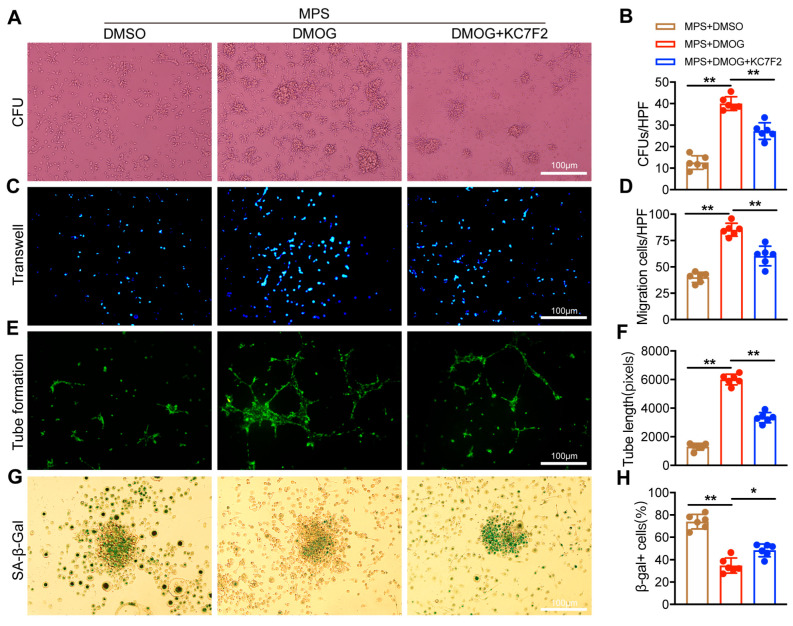
DMOG exerts a protective role in MPS-induced biological disfunctions via the HIF-1α signaling pathway in EPCs. (**A**,**B**) Representative images and quantitative analysis of CFU in EPCs (**C**,**D**) Transwell assay results of EPCs in different treatment groups. (**E**,**F**) Representative images and quantitative analysis of tube formation in EPCs treated with DMOG (500 μM) only or DMOG combined with KC7F2 (40 μM) for 24 h before MPS administration. (**G**,**H**) Representative images and quantitative analysis of SA-β-gal-positive EPCs for senescence detection. *n* ≥ six per group. * *p* < 0.05, ** *p* < 0.01. All data are presented as means ± SD. Statistical significance was determined by the one-way ANOVA with the post hoc Bonferroni’s test. Scale bar = 100 μm.

EPCs are essential for promoting vascular repair and regeneration owing to their stable biological functions [42]. To assess whether the effects of MPS and DMOG were mediated via the HIF-1α pathway, we first tested the colony formation ability of EPCs. The results showed that DMOG at 500 μM remarkably enhanced the CFU of EPCs compared to MPS, whereas the HIF-1α inhibitor significantly suppressed the ability to maintain colony formation in EPCs (Figure 3A,B). The migration ability of DMOG to EPCs was detected by the Transwell assay and nuclear staining. The results showed that MPS inhibited the migration of EPCs, whereas DMOG attenuated the adverse effect of MPS on EPCs. The loss of HIF-1α expression by KC7F2 (40 μM) disrupted these effects (Figure 3C,D). To further investigate the angiogenic potential of DMOG in EPCs, a Matrigel tube formation assay was performed in vitro. We observed that the tube structure of the DMOG group was richer than that of the MPS group, which had no loop formation. The total number of tubes, which indicated the start of vascular sprouting, was higher in the DMOG group, whereas the HIF-1α inhibitor KC7F2 compromised the normal tube structural conformation (Figure 3E,F). The development of senescence in vascular endothelial cells impairs normal vascular function and contributes to age-related vascular diseases [43,44]. Among β-galactosidase-positive cells, EPCs pretreated with DMOG showed a remarkably lower senescence rate than those treated with MPS; however, KC7F2 treatment led to a significantly higher proportion of senescence-associated cells (Figure 3G,H). On the basis of these results, we conclude that the activation of HIF-1α is highly effective against MPS-induced biological dysfunction in EPCs.

### 3.5. DMOG Reverses Osteonecrosis In Vivo

**Figure 4:**
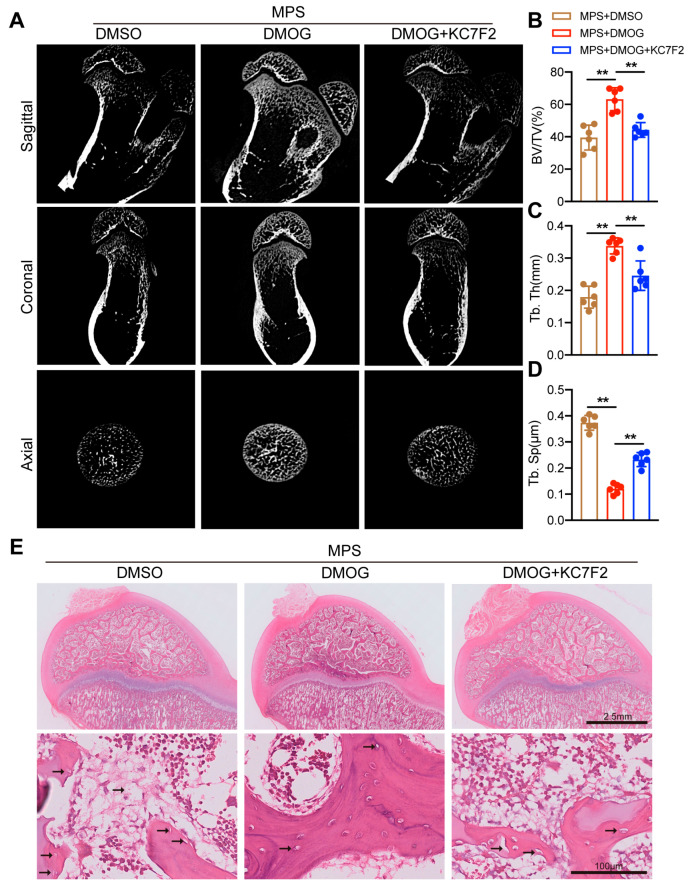
Histological staining of the femoral heads in rabbits. (**A**–**D**) Typical micro-CT images of the femoral heads. (**E**) Representative images of the subchondral bone of rabbit femoral heads in different groups. A large number of empty bone lacunae were observed next to the discrete bone trabecula in the MPS+DMSO group. The MPS+DMOG group exhibited fewer empty bone lacunae, and KC7F2 administration further aggravated osteonecrosis. Empty bone lacunae were shown by black arrows. ** *p* < 0.01. All data are presented as means ± SD. Statistical significance was determined by the one-way ANOVA with the post hoc Bonferroni’s test.

The success rate of MPS-induced ONFH in the MPS + DMSO group was 80% (8/10) 8 weeks after the first injection, whereas the incidence of osteonecrosis dropped to 30% (3/10) in the MPS + DMOG group. More pronounced osteonecrosis changes (6/10) were observed in the MPS-treated rabbits with KC7F2 administration before the initiation of DMOG injection as compared to the MPS + DMOG group. Micro-CT analysis revealed that MPS treatment led to substantial bone loss, as evidenced by the narrower trabecular bone and the remarkably increased trabecular separation (Tb. Sp) in the MPS group compared with those in the MPS + DMOG group, as well as strikingly decreased trabecular bone volume (BV/TV) and trabecular thickness (Tb, Th) (Figure 4A–D). Moreover, the total therapeutic state of the femoral heads was suppressed in the KC7F2-treated group compared with that in the DMOG-pretreated group (Figure 4A–D). H&E staining showed the diffuse presence of empty lacunae in the discrete trabecular bone (Figure 4E). The MPS + DMOG group showed more trabecular bone with fewer empty lacunae surrounded by adipocytes with a homogeneous shape. However, in the KC7F2 group, the number of empty bone lacunae was increased, compared to that in the MPS + DMOG group (Figure 4E).

### 3.6. DMOG Affects Angiogenesis and Bone Remodeling

**Figure 5:**
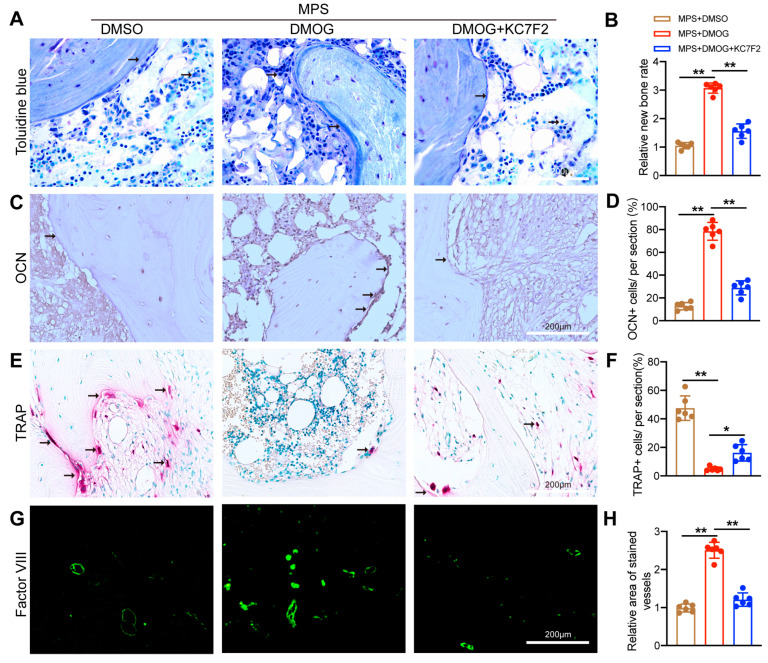
DMOG promotes osteogenesis and angiogenesis. (**A**,**B**) Toluidine blue and osteocalcin (**C**,**D**) staining of rabbit femoral heads in different groups. Comparison of new bone areas in different groups. New bone areas and osteoblasts were shown by black arrows. (**E**,**F**) TRAP staining of various groups indicated the process of bone resorption in the femoral heads. Bone resorption areas were shown by black arrows. (**G**,**H**) Immunofluorescence images and quantitative analysis of blood vessels in rabbit femoral heads. *n* ≥ six per group. * *p* < 0.05, ** *p* < 0.01. All data are presented as means ± SD. Statistical significance was determined by the one-way ANOVA with the post hoc Bonferroni’s test. Scale bar = 200 μm.

Toluidine blue staining was performed to detect osteogenesis in rabbit femoral heads. The results showed that obviously enlarged adipocytes accompanied by bone marrow cells with cytolysis were found in the MPS group. In addition, the formation of neonatal bone around necrotic bone trabeculae was observed after DMOG treatment, which indicated part of the repair process (Figure 5A,B). This therapy potential collapsed after pretreatment with KC7F2, which was further confirmed by osteocalcin staining (Figure 5C,D). Nevertheless, some studies have noted that HIF-1α activation is closely related to osteoclast activity [45,46]; therefore, TRAP staining was performed to detect osteoclast activity. Unexpectedly, activated HIF-1α did not affect osteoclast activity, whereas the HIF-1α inhibitor hindered this effect (Figure 5E,F). These results show that the HIF-1a pathway is not a significant factor associated with osteoclast activity. Furthermore, immunofluorescence staining was performed to assess angiogenesis in the necrotic area. The results revealed that DMOG enhanced the number of Factor Ⅷ-positive new vessels compared with MPS, whereas injection of an HIF-1α inhibitor attenuated the angiogenic response by DMOG (Figure 5G,H). In summary, HIF-1α pathway activation is required to partially alleviate MPS-induced osteonecrosis through its potential for angiogenesis and bone remodeling in vivo.

### 3.7. DMOG Increases the Numbers of EPCs Directional Migration in Rabbits Partially by Activating HIF-1α

**Figure 6:**
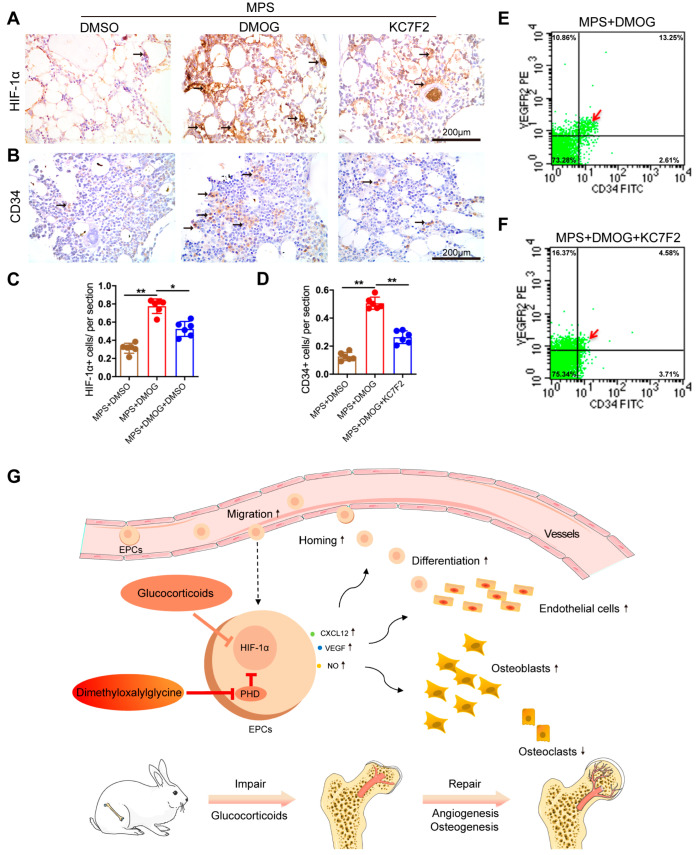
DMOG increases the level of HIF-1α and induces EPCs. (**A**,**C**) Immunohistochemical staining of rabbit femoral heads showed a higher level of HIF-1α in the MPS + DMOG group than in the MPS + DMSO group, whereas the MPS + DMOG + KC7F2 group showed significantly decreased HIF-1α expression. HIF-1α-positive cells were shown by black arrows. (**B**,**D**) Immunohistochemical staining of rabbit femoral heads revealed the number of CD34-positive cells in different groups. CD34-positive cells were shown by black arrows. (**E**,**F**) Flow cytometry detected EPCs in peripheral blood from the central ear artery in different groups. Double positive cells (CD34^+^/VEGFR2^+^) were considered to be EPCs (shown by red arrows). (**G**) Diagram showing how DMOG protects against osteonecrosis during glucocorticoids-induced endothelial damage in femoral heads. *n* ≥ six per group. * *p* < 0.05, ** *p* < 0.01. All data are presented as means ± SD. Statistical significance was determined by the one-way ANOVA with the post hoc Bonferroni’s test. Scale bar = 200 μm.

To further explore the potential role of DMOG and HIF-1α activation in steroid-induced osteonecrosis of the femoral heads, immunohistochemical staining for HIF-1α was performed. Compared with the MPS+DMSO group, DMOG significantly increased HIF-1α level in the femoral heads of MPS-treated rabbits. Nevertheless, a weaker HIF-1α signal surrounded by the microvascular embolization area in the femoral heads was observed in the KC7F2-pretreated group than that for the MPS + DMOG group (Figure 6A,C). In view of our finding that DMOG significantly upregulated HIF-1α in EPCs in vitro, we next sought to further explore the effects of DMOG on the mobilization of bone marrow-derived EPCs to a site of circulating peripheral blood as well as their targeting and homing to ischemic necrotic tissue, which contributed to the remodeling of injured blood vessels. Flow cytometry was performed to determine the number of circulating peripheral blood EPCs (CD34⁺/VEGFR2⁺ cells) from the central ear artery. To further purify the sorted endothelial progenitor cells, CD133 immunomagnetic beads were initially used for sorting [38]. The results revealed that the number of circulating EPCs from the central ear artery in rabbits significantly increased after DMOG treatment compared with that after treatment with HIF-1α inhibitor (Figure 6E,F). In addition, immunohistochemical staining of CD34⁺ cells in the femoral heads also revealed that, compared to that in the MPS group, the abundance of CD34⁺ cells was visible around the necrotic tissue of the femoral heads in the MPS + DMOG group, whereas the KC7F2 addition remarkably repressed the number of CD34⁺ cells in the femoral heads (Figure 6B,D). Taken together, these results show that directional migration of EPCs can be partially activated through HIF-1α activation after DMOG treatment, thereby facilitating the prevention of the progression of steroid-induced osteonecrosis of the femoral head.

## 4. Discussion

GCs are drugs widely used for autoimmune ailments because of their significant anti-inflammatory activity [47]. Nevertheless, more than 50% of GC users could progress to obvious bone loss, and approximately 40% of GC users could be faced with various degrees of osteonecrosis. The issue of long-term or excessive use of GCs, resulting in an increasing rate of SONFH, has been a health concern for many years. However, the etiology of SONFH has not been fully elucidated.

Currently, the vascular hypothesis of SONFH etiology seems to be the most convincing, postulating that impaired microvessels and reduced blood flow contribute to the occurrence and development of SONFH [48]. EPCs play an important role in postnatal neovascularization and function in the repair of injured blood vessels. For instance, transplantation of autologous EPCs can improve neovascularization and tissue repair [49]. Moreover, exosomes secreted by EPCs can significantly stimulate both angiogenesis and osteogenesis. Furthermore, PTEN inhibition may protect against steroid-induced EPC apoptosis by targeting the mitochondrial apoptosis pathway, thereby alleviating the development of SONFH [12]. Importantly, our previous findings also showed that the number and function of EPCs are impaired in SONFH patients [18,19]. Therefore, EPCs may serve as promising therapeutic targets for the prevention and treatment of SONFH.

HIF-1α is considered the most essential transcriptional regulator that mediates endothelial energy metabolism as well as cell proliferation and survival in response to hypoxia [50,51]. Recent research has demonstrated the important role of HIF-1α in the process of SONFH. For instance, activated HIF-1α in MSCs could not only promote VEGF secretion by the iron chelator deferoxamine required for maintaining PHD activity but also boost mitophagy and mitochondrial fission [28]. Scaffolds containing copper and iron significantly promoted the expression of HIF-1α and enhanced the number of MSCs homing into the femoral head, thereby repairing SONFH [30]. However, a previous study showed that HIF-1α is not an essential factor in the occurrence of SONFH [52]. It is possible that, despite HIF-1α upregulation, HIF-1α expression is relatively low at the late phase of osteonecrosis [53]. Intriguingly, several studies have revealed that endothelial cells undergo apoptosis and injure earlier than other bone cells, such as osteoblasts and MSCs, in response to glucocorticoid treatment [11,54]. These factors account for the likelihood of a potential therapy for SONFH via amelioration of endothelial dysfunction and vascular impairment. Therefore, it is necessary to further understand the relationship between HIF-1α and SONFH, particularly in EPCs.

Under normoxic conditions, HIF-1α is hydroxylated by PHD and then rapidly degraded through the ubiquitin-proteasome pathway; in low-oxygen conditions, decreased PHD activity attenuates the HIF-1α hydroxylation, thus resulting in an increase in HIF-1α translocation to the nucleus and the formation of heterodimerization with HIF-1β, which further activates the downstream signaling pathway. DMOG, an inhibitor of PHD, can stabilize HIF-1α and mimic true hypoxic conditions, which in turn participates in the repair of ischemic diseases [55]. Previous studies have shown the therapeutic potential of DMOG in osteoporosis and osteoarthritis [26,36]. Furthermore, DMOG have been widely used in bone tissue regeneration engineering because of their excellent bone repair capability, inducing both angiogenesis and osteogenesis [56]. Nevertheless, the effect of DMOG on SONFH has rarely been studied, especially in EPCs.

Our current study demonstrated that treatment with DMOG significantly increased the biological functions of EPCs. The cell viability and proliferative capability of EPCs were obviously enhanced by DMOG treatment at 50–500 μM in a time- and concentration-dependent manner. SDF-1 can improve endothelial cell mobilization, migration, homing, and angiogenesis partially via the SDF-1/ CXC chemokine receptor 4(CXCR4) axis under ischemic conditions [57]. In the present study, we found that DMOG increased the protein level of HIF-1α, accompanied by increased downstream target secretion of VEGF, SDF-1, and NO, which may promote the migration of EPCs through the bone marrow to the ischemic femoral head, thereby directly or indirectly contributing to the formation of new blood vessels. However, we showed that these effects could be attenuated by the HIF-1α inhibitor KC7F2. In addition, DMOG treatment not only promoted the chemotactic motility and colony-forming ability of EPCs, which is a direct indicator of reendothelialization and neovascularization, but also delayed EPCs senescence induced by MPS. Altogether, we proved that HIF-1α activation by DMOG is indispensable against MPS-induced impairment of EPCs by repressing the HIF-1α signaling pathway by KC7F2 in vitro.

The impaired function of vascular endothelial cells caused by GCs is believed to be an essential cause of SONFH [18]. Our in vivo data were consistent with those of previous studies, which revealed that MPS injection significantly induced osteonecrosis in the femoral heads of rabbits, as evaluated by H&E staining and micro-CT. Nevertheless, the damaged microvessels could be alleviated after DMOG injection therapy in rabbit SONFH models. Immunofluorescence analysis also revealed that DMOG induced a significant increase in the number of neo-vessels. Toluidine blue and osteocalcin staining showed that DMOG promoted osteogenesis in the femoral heads. These findings are in sharp contrast to the large number of empty bone lacunae observed following MPS treatment. Mobilization, migration, and homing of EPCs to the site of vascular injury during the early process are important to facilitate tissue repair [58]. Our study showed that activation of HIF-1α by DMOG significantly enhanced the number of EPCs in the femoral head. Nevertheless, pretreatment with an HIF-1α inhibitor significantly disrupted the protective effects of DMOG on MPS-induced osteonecrosis in rabbits. Notably, although many studies have pointed out that the activation of HIF-1α could be associated with osteoclastogenesis and osteoclast activity, the remarkably increased bone-resorbing activity and decreased HIF-1α expression in the necrotic area indicated that HIF-1α might not be the main factor regulating bone-resorbing cells in SONFH. These could be exemplified by some recent studies on how GCs inhibit preosteoclasts and osteoclast activity, suggesting that other factors, such as oxidative stress, contribute to the bone-resorbing process in SONFH [59,60,61]. DMOG treatment might alleviate the effects of oxidative stress and other factors stimulated by GCs. Further studies on the relationship between DMOG and the bone-resorbing process are warranted. In conclusion, our findings confirm that targeting the activation of HIF-1α in EPCs by DMOG might be a promising therapy for SONFH.

In the present study, we demonstrated the protective effects of DMOG against MPS-induced osteonecrosis of the femoral head in rabbits. Our in vitro and in vivo studies confirmed that HIF-1α activation by DMOG may play a highly significant role in SONFH. However, there are some limitations to the present study. For instance, the mechanisms of HIF-1α activation are limited to its downstream signaling molecules SDF-1, VEGF, and NO induced by DMOG. Further experiments on these potential mechanisms targeting the HIF-1α signaling pathway might be of great value. In addition, owing to the incompletely consistent mechanisms between rabbit- and human-derived EPCs, further clinical samples of EPCs should be conducted to better account for DMOG effects in our studies.

## 5. Conclusions

In conclusion, we demonstrated that high doses of GCs could suppress HIF-1α expression in EPCs, resulting in impaired biological functions both in vivo and in vitro, whereas the addition of DMOG alleviated these concerns and stimulated the mobilization and homing of EPCs into the femoral head. However, these effects were reversed by treatment with the HIF-1α inhibitor KC7F2, which further supports the importance of the HIF-1α signaling pathway in SONFH. Hence, activation of the HIF-1α signaling pathway by DMOG could be a promising EPC-based therapeutic strategy for the prevention and development of SONFH.

## Data Availability

The data that support the findings of this study are available from the corresponding author upon reasonable request.
